# Essential updates 2018/2019: Colorectal (benign): Recent updates (2018‐2019) in the surgical treatment of benign colorectal diseases

**DOI:** 10.1002/ags3.12304

**Published:** 2019-12-16

**Authors:** Takayuki Ogino, Tsunekazu Mizushima, Chu Matsuda, Masaki Mori, Yuichiro Doki

**Affiliations:** ^1^ Department of Gastroenterological Surgery Graduate School of Medicine Osaka University Osaka Japan; ^2^ Department of Therapeutics for Inflammatory Bowel Diseases Graduate School of Medicine Osaka University Osaka Japan; ^3^ Department of Surgery and Science Graduate School of Medical Sciences Kyusyu University Fukuoka Japan

**Keywords:** benign colorectal disease, Crohn's disease, diverticulitis, laparoscopic surgery, ulcerative colitis

## Abstract

This review outlines current topics on the surgical treatment of benign colorectal diseases, with a focus on inflammatory bowel disease (IBD) and diverticulitis. Treatment options for IBD and diverticulitis have evolved in the last few years as a result of medical advances in technology and new clinical trials. Therefore, treatment options and strategies need to be updated to provide optimal care for patients. The purpose of this review is to elucidate recent global trends and update the surgical treatment strategy for IBD and diverticulitis based on literature published in the past 2 years. Prevalence of IBD, including ulcerative colitis and Crohn's disease, has increased over the last few decades. During this period, many new medical therapies were introduced for the treatment of IBD, including biological therapy, immunomodulators, and leukocyte apheresis therapy. As a result, new surgical strategies for IBD are required. In order to improve surgical outcomes in IBD patients, the influence of preoperative treatment on postoperative complications needs to be considered. The incidence of diverticulitis is also increasing with lifestyle changes and increasing numbers of older people. For diverticulitis with perforation and generalized peritonitis, surgery is the gold standard. Elective surgery after conservative treatment of diverticulitis is also an option because of high recurrence rates. With an increase in diverticulitis, systematic strategies are essential for an appropriate approach to diverticulitis, taking into account various factors, including the patient's background.

## 1 INTRODUCTION

In recent decades, the number of patients with inflammatory bowel disease (IBD) and diverticulitis has dramatically increased in developed countries.^1^, ^2^ Treatment options for IBD and diverticulitis have evolved over the last few years due to medical advances in technology and new clinical trials. Therefore, treatment options and strategies need to be updated to provide optimal care for patients. IBD refers to two distinct forms of disease, ulcerative colitis (UC) and Crohn's disease (CD), which are characterized by relapsing and remitting conditions and chronic inflammation in the intestine.^3^, ^4^ Development and/or pathogenesis of IBD is considered to be an inadequate immune response to luminal contents. New medical therapies have been rapidly introduced for the treatment of IBD, such as biological therapy, immunomodulators, and leukocyte apheresis therapy, among others.^5^, ^6^ Surgical treatments corresponding to these changes are also needed. In contrast, a diverticulum is a small outpouching from the intestinal lumen due mostly to mucosal herniation through the wall at sites of vascular perforation.^2^, ^7^ Diverticulitis is inflammation or infection of the diverticulum, which occurs mostly in the colon. In Japan, diverticulosis is increasing because of a widespread elderly population and changing lifestyle. Approximately 80% of patients with diverticulosis remain asymptomatic, and the other 20% of patients develop diverticulitis, requiring medical treatment.^2^

It is expected that the needs for surgical treatment of IBD and diverticulitis will increase in the near future with the increase in the elderly and prevalence. The present review highlights recent global trends and updates to surgical treatment strategies in IBD and diverticulitis based on the literature published in the last 2 years (2018‐2019). Several important studies are referred to as necessary information for surgeons. To facilitate understanding of the background of each procedure, papers published before 2017 were reviewed when applicable.

## 2 INFLAMMATORY BOWEL DISEASE

Inflammatory bowel disease is a chronic disease that causes unexplained inflammation in the gastrointestinal tract and comprises UC and CD. The number of patients is increasing globally, as well as in Japan.^8^, ^9^ Abnormalities in the gut immune system are thought to be highly involved in the development of IBD, but the exact pathogenic mechanism is unclear.^2^, ^8^ As both UC and CD often occur in young people and require long‐term treatment, they not only lower the quality of life (QOL), but hinder social activities, such as schooling, work, marriage, and childbirth. In addition, new problems, such as inflammation‐related carcinogenesis, have emerged with an increase in long‐term cases.^10^

Biological therapy based on disease mechanisms appeared in the 2000s. Patients’ QOL improved, and both medical treatment and surgical treatment changed significantly. A study of US patients between 2009 and 2015 showed that the use of biological therapy increased from 20% to 40% in CD patients, and from 5% to 16% in UC patients.^11^ Kimura et al^12^ showed that in 2011, Japanese patients treated with a biological preoperatively increased dramatically, and that in 2013, 41% of UC patients who underwent surgery had received biological treatment. Japanese nationwide cohort study also showed the rate of administration of anti‐tumor necrosis factor (TNF) increased from 0.3% in 2007 to 43% in 2017 among UC patients who underwent restorative proctocolectomy.^13^ Given the continuous emergence of biological therapies used more frequently in severe IBD, we are in a new era of biological therapy, including anti‐TNFα, anti‐interleukin (IL)‐12/23p40, anti‐integrin α4β7, and Janus kinase inhibitor, which will likely continue for some time. Assessment of variability in real‐world practice is essential to optimize the timing of initial therapy and surgery for IBD patients. According to a study of regional differences in the treatment of IBD after 2006, 66% of CD and 28% of UC patients in the USA commonly used biological therapy, compared to 19% of CD and 0% of UC patients in China. No differences were seen in the proportion of patients undergoing early surgery.^14^

With regard to surgical treatment, preoperative conditions in IBD are often immunosuppressive or patients are undernourished, and different from other bowel diseases, such as colorectal cancer. This section outlines points to be aware of in the surgical treatment of UC and CD.

### 2.1 Surgical treatment and biological therapy

Several reports, including randomized controlled trials, assessed preoperative treatment and surgical rates. The Active Ulcerative Colitis Trial (ACT) reported the efficacy of infliximab for induction and maintenance therapy and the cumulative incidence of colectomy in 728 patients with moderate‐to‐severe active UC. Patients receiving infliximab had a decreased Mayo score with decreased rectal bleeding compared to placebo patients. At 54 weeks of follow up, the colectomy rate was 10% in the infliximab group and 17% in the placebo group; which means the absolute risk of colectomy decreased by 7%.^15^, ^16^ Another study evaluated the short‐ and long‐term efficacy of infliximab in 45 patients with steroid‐resistant UC (24 infliximab and 21 placebo); 29% in the infliximab group and 67% in the placebo group underwent colectomy within 3 months, and 50% in the infliximab group and 76% in the placebo group within 3 years. No patient death was reported. Patients who had endoscopic remission within 3 months did not require colectomy, even after 3 years. The main benefit of infliximab occurred within the first 3 months, and early mucosal healing reduced the risk of subsequent colectomy.^17^, ^18^ The CONSTRUCT study showed the efficacy of infliximab and cyclosporine in 270 patients with steroid‐resistant UC. The colectomy rate within 3 years was 41% in the infliximab group and 48% in the cyclosporine group, and no significant differences were observed between the two groups.^19^ Laharie et al also reported the colectomy rates within 3 months for 115 patients with steroid‐resistant UC: 21% in the infliximab group and 17% in cyclosporine group. The 5‐year colectomy‐free survival rate was not different between the infliximab group and the cyclosporine group (65% vs 62%, respectively). Death directly related to UC or treatment was not observed.^20^, ^21^ A meta‐analysis showed short‐term clinical response rates in 72.1%, clinical remission rates in 52.4%, and 3‐month colectomy rates in 10.1% among patients receiving tacrolimus with moderate‐to‐severe and steroid‐refractory UC. No significant difference was seen for tacrolimus compared with anti‐TNF with regard to clinical remission rate, clinical response rate, and 3‐month colectomy rate.^22^ Narula et al reported the efficacy of anti‐TNF agents and calcineurin inhibitors including tacrolimus and cyclosporine for 314 patients with severe steroid‐refractory UC. Patients with sequential treatment achieved short‐term response in 62.4% and remission in 38.9%; however, the colectomy rates were high with 28.3% at 3 months and 42.3% at 12 months.^23^ Takeuchi et al^24^ also showed that tacrolimus and infliximab were equally effective in short‐term clinical remission and response rates, and in colectomy‐free rates for active UC.

Difference between the surgical approach and postoperative outcome was studied in 7070 patients with UC before (1995‐2005) and after the introduction of biological therapy (2005‐2013). The proportion of patients who underwent at least three procedures increased significantly in the post‐introduction group. Major events, procedural complications, and non‐routine discharge were also observed in the post‐introduction group.^25^ In Japan, reoperation rate in CD patients significantly decreased after the introduction of anti‐TNF agents in 2002. Risk factors for reoperation were preoperative smoking, perianal disease, and ileocolic‐type CD.^26^ A survey of UC patients from 2008 to 2013 reported the relationship between the introduction of biological therapy and surgery. The number of patients undergoing surgery decreased immediately after the introduction of infliximab and tacrolimus, but then increased again. Emergency surgery rates did not change throughout the study period.^12^

### 2.2 Surgical treatment

When emergency subtotal colectomy is carried out, we usually select i.p. placement of the closed rectal stump in order to prevent inflammatory adhesion at the next remnant rectal resection. Bedrikovetski et al carried out a systematic review of the appropriate management of rectal stumps after emergency subtotal colectomy in patients with acute severe UC. A total of 476 patients were assessed regarding closed s.c. placement of the rectal stump, i.p. placement, or mucous fistula formation. Pelvic sepsis rates were lowest (2%) in patients with s.c. placement. Patients with i.p. placement had less wound infection but high mortality.^27^ Risk factors in patients with chronic refractory UC were an absence of clinical response and lack of mucosal healing after induction with biological therapy. Early assessment (12‐16 weeks after therapy) of the clinical and endoscopic response could predict subsequent risk of colectomy.^28^

In CD, perianal lesions relapse the same as intestinal lesions, and exacerbation of lesions or inappropriate surgical treatment leads to decreased anal function and QOL. Selection of appropriate treatment is necessary for surgeons to maintain anal function. In 15 CD patients with complex perianal fistula, efficacy, safety, and feasibility of local administration of microfragmented adipose tissue were reported. During 24 weeks of follow up, 10 patients had remission, four patients improved, and one patient failed. No relevant postoperative complications or adverse events were observed. This procedure was minimally invasive with little risk of sphincter damage.^29^

With progress in medical treatment for IBD, surgical indications and prognosis have changed. The treatment effect should be evaluated early, and surgical treatment should be carried out before the general condition worsens without continuing with inadequate medical treatment for a long period of time.

### 2.3 Postoperative complications

Crohn's disease cannot be completely cured by surgery, and redo surgery for recurrence is often necessary. For efficient prevention of postoperative recurrence, it is essential to identify high‐risk cases of recurrence. The relationship between preoperative biological therapy and postoperative complications has been studied, but is still controversial. Gutiérrez et al studied early postoperative complications in 364 adult CD patients who underwent ileocolectomy with anastomosis; complications were observed in 27.5% of patients, mainly wound infections, intra‐abdominal abscesses, and anastomotic leakages. Complications were more common in patients with penetrating disease and those refractory to treatment, and urgent surgeries were associated with an increased risk of complications.^30^ Postoperative recurrence is a major problem in CD patients after ileocolectomy. Allez et al conducted a T‐cell receptor analysis of surgical specimens in 57 CD patients; clonal T‐cell expansion was associated with smoking. Clonal T‐cell expansion was also implicated in postoperative endoscopic recurrence, and highly clonal patients showed increased expression of genes related to CD8+ T cells.^31^ Introduction of immune cell evaluation would be key for appropriately predicting recurrence.

The effect of vedolizumab, a selective intestinal monoclonal antibody, on postoperative complications is still unknown. Law et al evaluated the impact of preoperative vedolizumab treatment on postoperative complications in IBD patients undergoing abdominal surgery. This systematic review included 307 patients in the vedolizumab‐treated group, 490 patients in the anti‐TNF treatment group, and 535 patients who did not receive preoperative biological therapy; preoperative vedolizumab treatment was not associated with an increased risk of postoperative complications compared to preoperative anti‐TNF treatment or no biological therapy in IBD patients.^32^ Yung et al reported that the risk of postoperative complications was not significantly different between preoperative vedolizumab and anti‐TNF in IBD patients. Particularly in UC patients, the risk of overall postoperative complications was lower in the vedolizumab group.^33^ Novello et al compared postoperative complications in 30 CD patients treated with ustekinumab and 73 patients treated with vedolizumab prior to colorectal surgery within 12 weeks. Choice of preoperative biologic therapy, ustekinumab or vedolizumab, did not influence postoperative complications.^34^ Another case‐matched analysis showed that exposure to preoperative vedolizumab was not associated with increased morbidity, but the majority of patients had an ostomy.^35^ The impact of biologicals on postoperative complications is still controversial. Summary of previous reports is shown in Table 1.^23^, ^30^, ^32^, ^33^, ^34^, ^35^, ^36^, ^37^, ^38^, ^39^, ^40^, ^41^, ^42^ Large prospective studies are required to draw conclusions.

**Table 1:** Impact of biologicals on postoperative complications

Author	Year	Disease	No. patients	Biologicals	Postoperative complications
Kopylov et al^36^	2012	CD	1641	TNF vs no Bio	Increased risk of postoperative complications
Billioud et al^37^	2013	IBD	4251	TNF vs no Bio	Increased risk of infectious complications in CD, Not associated with UC
Narula et al^23^	2015	IBD	4659	TNF vs no Bio	Increased risk of postoperative complications
Lau et al^38^	2015	UC	94	TNF vs no Bio	Not associated
Yamada et al^39^	2017	IBD	443	VEDO vs TNF vs no Bio	Not associated
Fumery et al^40^	2017	CD	209	TNF vs no Bio	Not associated
Kulaylat et al^41^	2017	UC	2476	TNF vs no Bio	Increased risk of postoperative complications in IPAA
Lightner et al^42^	2017	UC	146	VEDO vs TNF	Increased risk of SSI in VEDO
Law et al^32^	2018	UC	1332	VEDO vs TNF vs no Bio	Not associated
Yung et al^33^	2018	IBD	1080	VEDO vs TNF vs no Bio	Not associated
Novello et al^34^	2019	CD	103	UST vs VEDO	Not associated
Novello et al^35^	2019	UC	980	VEDO vs no Bio	Increased risk of postoperative complications in VEDO
Novello et al^35^	2019	UC	980	VEDO vs TNF	Not associated
Gutiérrez et al^30^	2019	CD	364	TNF vs no Bio	Not associated

Abbreviations: Bio, biologicals; TNF, anti‐tumor necrosis factor (TNF) agent; UST, ustekinumab; VEDO, vedolizumab.

Inflammatory bowel disease is associated with a 1.5‐ to 3‐fold increased risk of venous thromboembolism (VTE). Sarlos et al reported the risk of VTE during corticosteroid or anti‐TNFα therapy in 58 518 patients with IBD. VTE events occurred in 5.6% of patients. The corticosteroid group had a significantly higher incidence of VTE. In contrast, anti‐TNFα therapy had a fivefold lower risk of VTE compared to corticosteroids.^43^ Benlice et al reported the risk factors for 30‐day VTE from an analysis of 24 182 IBD patients after elective abdominopelvic bowel surgery. The 30‐day total and post‐discharge rates of VTE were 2.5% and 1%, respectively. Risk of VTE was associated with older age, steroid use, bleeding disorders, open surgery, hypertension, longer operative time, preoperative hospitalization, postoperative transfusion, and pelvic enterocutaneous fistula surgery.^44^

Hypoalbuminemia is a prognostic factor for postsurgical outcomes. Nguyen et al studied its role in predicting postoperative outcomes in 6082 CD and 4831 UC patients who underwent bowel surgery. Hypoalbuminemia was related to 30‐day mortality and infectious complications in both CD and UC patients, and was associated with extra‐intestinal complications, such as postoperative bleeding, cardiac failure, neurological failure, failure to wean off ventilators, VTE, and reoperation within 30 days.^45^

### 2.4 Positioning of surgical treatment

The LIR!C trial evaluated the cost‐effectiveness of laparoscopic ileocecal resection compared to infliximab in CD patients who failed more than 3 months of conventional immunomodulator or steroid therapy without signs of critical strictures. A total of 143 patients were included in this randomized trial, and total direct healthcare and social costs were lower in the resection group than in the infliximab group. Laparoscopic ileocecal resection is a cost‐effective treatment compared to infliximab.^46^ Murthy et al evaluated the impact of infliximab on hospitalization, surgery rates, and costs in IBD patients living in Ontario, Canada. The introduction of infliximab did not result in a significant reduction in hospitalization and surgery rates among CD patients, whereas the hospitalization rates declined substantially among UC patients. They reported a threefold increase in drug costs for CD patients following the introduction of infliximab, but no significant change in UC patients.^47^

The CONSTRUCT study showed the use of cyclosporine led to lower total costs compared to infliximab in UC patients. Nevertheless, no significant difference was found between these drugs regarding clinical effectiveness, colectomy rates, incidence of side‐effects, or mortality 1‐3 years post‐treatment. However, participants were more positive about infliximab than cyclosporine, and nurses disliked the i.v. cyclosporine. 19

In recent years, enhanced recovery after surgery (ERAS) has been shown to reduce length of hospital stay, complications, and costs after colorectal surgery, but the effect on IBD has been unclear. Liska et al reported an improvement in outcomes using ERAS in 671 IBD patients. Implementation of ERAS for IBD patients resulted in a decrease in length of hospital stay and costs without any increase in complications and readmissions.^48^

Robotic surgery for IBD has gradually spread, but the hybrid approach would currently be optimal for complicated cases.^49^ Mizushima et al^50^ reported that single‐incision laparoscopic surgery can be carried out safely in patients with stricturing or penetrating CD. In UC patients undergoing ileal pouch‐anal anastomosis, the 30‐day postoperative complication rate was comparable to laparoscopic surgery.^51^ The use of open, laparoscopic, and robotic surgery should be balanced with cost‐effectiveness and postoperative outcomes.

### 2.5 Bariatric surgery for IBD patients

In recent years, the relationship between obesity and IBD has attracted attention. Cañete et al studied the impacts of bariatric surgery on IBD. After bariatric surgery, 17 patients developed UC, 60 CD, and three unclassified IBD. Female gender (82%) was predominant, median age was 45 years, median BMI before surgery was 47 kg/m^2^, and 80% of bariatric surgery techniques were Roux‐en‐Y gastric bypass (RYGB). Potentially IBD‐related symptoms occurred within 1 month to 16 years after the surgery. Twenty‐four patients with UC, 35 patients with CD, and one patient with unclassified IBD underwent bariatric surgery. Sleeve gastrectomy (SG) was the most frequent technique and could be the procedure of choice in these patients.^52^ Heshmati et al showed the impacts of bariatric surgery in 31 CD patients and 23 UC patients; 19 patients underwent RYGB and 35 SG. There was a significant difference in the proportion of patients who had worsened CD after RYGB compared to SG (37.5% vs 4%). SG resulted in less weight loss but lower surgical complications compared to RYGB (26% vs 3%). In patients with IBD, especially CD, SG may be a safer surgical technique.^53^

## 3 DIVERTICULITIS

Diverticulitis of the colon is increasing in developed countries as a result of adaption of a Western lifestyle and an increased elderly population. Computed tomography (CT) is a useful method for diagnosing diverticulitis.^7^ Non‐complicated diverticulitis and diverticulitis with localized abscess are usually managed with conservative treatment. However, surgery or percutaneous drainage should be considered in a case with resistance to conservative treatment. Surgery is selected mainly for diverticulitis with perforation and generalized peritonitis.^2^, ^7^ Because recurrence of diverticulitis often occurs after conservative treatment, elective surgery should be considered. Herein, we describe the surgical treatment for diverticulitis.

### 3.1 Surgical approach

Safety and effectiveness of laparoscopic surgery for diverticulitis have been reported in recent years. According to a case‐control matching study, there was no significant difference in the complication rate, reoperation rate, readmission rate, and mortality between open and laparoscopic surgery. Laparoscopic surgery resulted in a shorter hospital stay and improved postoperative outcomes in patients with preoperative respiratory comorbidities. Between open and laparoscopic surgery for acute diverticulitis, no significant difference was shown in postoperative morbidity and mortality during short‐ or long‐term follow up.^54^, ^55^, ^56^ For diverticular disease, single‐incision laparoscopic surgery was equivalent to open sigmoidectomy regarding complications, but resulted in less pain, fewer blood transfusions, and shorter length of hospital stay.^57^

The use of robotic surgery for colorectal diseases has been reported in recent years. Ogilvie et al studied elective sigmoidectomy for diverticulitis, comparing laparoscopic and robotic surgery. Sixty‐nine robotic cases were propensity‐matched from a group of 222 laparoscopic cases; they found no difference in postoperative pain and length of stay, but total hospital costs were $15 000 higher for robotic surgery.^58^ Robotic and laparoscopic surgery were also compared in the elective management of left side diverticulitis. Robotic surgery was associated with shorter hospital stay (3.89 vs 4.75 days), lower conversion rate (7.5% vs 14.3%), and longer operative time (219.2 vs 188.8 minutes) than laparoscopic surgery.^59^ Cassini et al evaluated the effectiveness, potential benefits, and short‐term outcomes of 64 patients undergoing robotic surgery compared to 92 patients undergoing laparoscopic surgery for complicated diverticulitis. No conversions occurred in the robotic group compared to a 6.5% conversion rate in the laparoscopic group. Operative time, blood loss, hospital stay, and postoperative morbidity were not significantly different between the two groups.^60^ Raskin et al^61^ showed that the robotic‐assisted approach was associated with fewer conversions to an open approach, shorter hospital length of stay, fewer postoperative complications (ileus, wound complications, and acute renal failure), and more patients discharged directly to home compared to laparoscopic and open approaches.

However, most of these studies were reported from experienced facilities. The indication should be carefully determined by taking into account the skill of the surgeon and the patient's condition. Yeom et al reported the outcomes of laparoscopic surgery in patients with pan‐peritonitis. Postoperative complications occurred in 21.6%, and mortality in 4.8%. Preoperative shock (<90 mm Hg) and a longer time from symptom onset (over 2 days) to surgery were prognostic factors for postoperative mortality.^62^ Therefore, careful patient selections are necessary for laparoscopic surgery. Open surgery should be considered in cases with long duration from onset, cases with shock, cases with serious comorbidities, and/or cases with pan‐peritonitis.

### 3.2 Surgical procedure

Hartmann's operation has been carried out conventionally for complicated diverticulitis. The Hartmann operation has the challenge of stoma reversal and 30%‐40% of stoma cannot be closed.^63^, ^64^ Primary resection and anastomosis, and laparoscopic lavage are widespread as an alternative surgery. The LADIES trial assessed outcomes after Hartmann's procedure versus sigmoidectomy and primary anastomosis with or without protective ileostomy in 133 patients with severe sigmoid diverticulitis (Hinchey III or IV disease) aged <85 years. Twelve‐month stoma‐free survival was significantly better in patients with primary anastomosis, and no significant differences were observed in short‐term morbidity and mortality between the two procedures.^65^

Several studies have reported that primary anastomosis was similar to Hartmann's operation regarding major postoperative complications, mortality, and readmission rate.^66^, ^67^ However, Cauley et al^68^ reported that complication rates and in‐hospital mortality rates for primary anastomosis with diversion were higher than those for Hartmann's procedure. According to a review by Cirocchi et al, there is no significant difference in mortality and overall morbidity between primary anastomosis and Hartman's operation for perforated sigmoid diverticulitis with generalized peritonitis, although postoperative intra‐abdominal abscesses are fewer after primary anastomosis. Permanent stoma rates were not significantly different in these groups.^69^ Goldstone et al reported that postoperative mortality was twofold greater when general surgeons carried out a primary anastomosis compared to Hartmann's operation (7.4% vs 15%). Primary anastomosis by general surgeons was associated with postoperative complications and reoperation, whereas colorectal board certification was associated with decreased mortality.^70^

Several studies of laparoscopic lavage as an alternative procedure have been reported in recent years.^71^ The DILALA trial reported outcomes after laparoscopic lavage versus open Hartmann's procedure in patients with Hinchey grade III perforated diverticulitis. The proportion of patients who underwent one or more secondary operations within 24 months was lower in the laparoscopic lavage group (41.8%) compared to the Hartmann's procedure group (67.5%). The authors reported no difference in readmissions or mortality between these procedures.^72^ Penna et al also studied clinical outcomes after laparoscopic lavage or colonic resection in 589 patients with purulent diverticulitis. They reported no significant differences in mortality, 30‐day reoperation, or unplanned readmissions. The laparoscopic lavage group had more intra‐abdominal abscesses, peritonitis, and long‐term emergency reoperations, but this group had shorter operative time, fewer cardiac complications, fewer wound infections, and shorter hospital stay; 14% of patients in this group required a stoma.^73^ However, some reports found an association between laparoscopic lavage and increased morbidity, whereas laparoscopic lavage and other surgical procedures had comparable rates of early reoperation and postoperative mortality.^74^ Sneiders et al reported outcomes in patients treated with laparoscopic peritoneal lavage without sigmoidectomy for perforated diverticulitis with purulent peritonitis. More than 30% required additional surgery and readmissions; 31% of patients initially treated successfully had recurrent diverticulitis or other complications, and 22% of these patients eventually had a sigmoidectomy within 90 days.^75^

After Hartmann operation for diverticulitis requiring surgery, optimal timing of subsequent colostomy reversal remains unknown. Resio et al reported that early reversal (45‐110 days) is associated with patient age (≤60 years), ethnicity (Caucasian), and private insurance. Prolonged length of stay and 90‐day readmissions were significantly increased with late reversal, whereas mortality, transfusion, ileus, and major complications were not significantly associated with reversal timing.^76^ Open surgery, preoperative steroid use, and disease‐related factors were involved in ileostomy creation after primary anastomosis.^77^ Surgery for uncomplicated diverticulitis was also reported. Luu et al reported that both laparoscopic diverticulectomy and non‐operative treatment were safe and effective in patients with uncomplicated right‐sided colonic diverticulitis. Laparoscopic diverticulectomy could be an option in a case with possible recurrence.^78^

### 3.3 Postoperative complications and long‐term outcomes

The DIRECT trial showed significantly better QOL (less pain, lower risk of new recurrences) at the 5‐year follow up in patients who underwent elective sigmoidectomy compared to conservative treatment for recurring diverticulitis and/or ongoing complaints after an episode of diverticulitis. Forty‐six percent of patients with conservative treatment required surgery as a result of severe ongoing complaints.^79^ This trial also showed that elective sigmoidectomy is cost‐effective compared to conservative treatment.^80^

Risk factors and postoperative outcomes were evaluated in patients who underwent surgery for diverticulitis. Emergency surgery was associated with worse preoperative conditions and more postoperative complications, including mortality. Patients with comorbid conditions may be a better population for elective colectomy.^81^ An et al^54^ reported that preoperative serum albumin <3.0 g/dL affected the mortality rate. Varma et al retrospectively studied the timing of surgery in 4478 patients with an initial episode of uncomplicated diverticulitis followed by a bowel resection within 2 years. One‐fifth of patients underwent emergency resection, and median time from the initial episode to resection was 3.8 months for elective resections and 5.1 months for emergency resections. The odds of having an emergency surgery increased with every three passing months. Emergency surgery was also associated with more postoperative complications, 30‐day readmissions, and longer length of hospital stay.^82^ Lambrichts et al assessed the outcomes of non‐surgical treatment and identified risk factors for adverse outcomes in 447 patients with Hinchey Ib or II diverticular abscess. Treatment strategy, percutaneous drainage with antibiotics versus antibiotics alone, was not associated with short‐term treatment failure, emergency surgery, or long‐term surgery. Abscesses more than 3 cm were associated with short‐term treatment failure, and abscesses more than 5 cm were associated with the need for surgery.^83^

After surgery for diverticulitis, patients with metabolic syndrome (BMI >30 kg/m^2^, hypertension, and DM) had more adverse events, such as reintubation, ventilator dependence more than 48 hours, myocardial infarction, and superficial or deep surgical site infections. Patients with metabolic syndrome also had longer recovery and higher rates of complications, readmissions, and mortality.^84^ Bordeianou et al reported that 21% of patients with diverticulitis had surgical site infection. Obesity (BMI >30 kg/m^2^), advanced age (>70 years), diabetes mellitus, preoperative abscess, open surgery, emergency operations, and prolonged operations (>3 hours) were predictors of infection.^85^ Al‐Temimi et al compared surgically managed right side and left side diverticulitis. Patients with right side diverticulitis were more likely to be Asian and had a higher BMI than those with left side diverticulitis. Surgery for right side diverticulitis was associated with shorter hospital stay and less diverting stoma, but postoperative complications were not significantly different between right and left side disease.^86^

## 4 CONCLUSIONS

In the present review, we updated advancements in the surgical treatment of IBD and diverticulitis based on recent findings. The prevalence of these diseases will increase in the future as already seen in developed countries. Although surgical technology, including robotic surgery, is rapidly progressing, surgeons need to carry out the most appropriate treatment to prevent unfavorable outcomes for patients. Not only colorectal surgeons, but also general surgeons, should always keep in touch with these novel ideas and concepts to improve the QOL of patients.

## DISCLOSURE

Conflicts of Interest: Authors declare no conflicts of interest for this article.

## References

[1] Kaplan GG . The global burden of IBD: from 2015 to 2025. Nat Rev Gastroenterol Hepatol. 2015;12:720–7. 26323879 10.1038/nrgastro.2015.150

[2] Stollman N , Raskin JB . Diverticular disease of the colon. Lancet. 2004;363:631–9. 14987890 10.1016/S0140-6736(04)15597-9

[3] Xavier RJ , Podolsky DK . Unravelling the pathogenesis of inflammatory bowel disease. Nature. 2007;448:427–34. 17653185 10.1038/nature06005

[4] MacDonald TT , Monteleone I , Fantini MC , Monteleone G . Regulation of homeostasis and inflammation in the intestine. Gastroenterology. 2011;140:1768–75. 21530743 10.1053/j.gastro.2011.02.047

[5] Hindryckx P , Vande Casteele N , Novak G , Khanna R , D'Haens G , Sandborn WJ , et al. The expanding therapeutic armamentarium for inflammatory bowel disease: how to choose the right drug(s) for our patients? J Crohns Colitis. 2017;12:105–19. 10.1093/ecco-jcc/jjx117 28961959

[6] Masaki T , Kishiki T , Kojima K , Asou N , Beniya A , Matsuoka H . Recent trends (2016–2017) in the treatment of inflammatory bowel disease. Ann Gastroenterol Surg. 2018;2(4):282–8. 30003191 10.1002/ags3.12177 PMC6036397

[7] Tochigi T , Kosugi C , Shuto K , Mori M , Hirano A , Koda K . Management of complicated diverticulitis of the colon. Ann Gastroenterol Surg. 2017;2(1):22–7. 29863123 10.1002/ags3.12035 PMC5868871

[8] Mandel MD , Miheller P , Mullner K , Golovics PA , Lakatos PL . Have biologics changed the natural history of Crohn's disease? Dig Dis. 2014;32:351–9. 24969279 10.1159/000358135

[9] Murakami Y , Nishiwaki Y , Oba MS , Asakura K , Ohfuji S , Fukushima W , et al. Estimated prevalence of ulcerative colitis and Crohn's disease in Japan in 2014: an analysis of a nationwide survey. J Gastroenterol. 2014;54(12):1070–77. 10.1007/s00535-019-01603-8 31309327

[10] Paramsothy S , Rosenstein AK , Mehandru S , Colombel JF . The current state of the art for biological therapies and new small molecules in inflammatory bowel disease. Mucosal Immunol. 2018;11:1558–70. 29907872 10.1038/s41385-018-0050-3 PMC6279599

[11] Yu H , MacIsaac D , Wong JJ , Sellers ZM , Wren AA , Bensen R , et al. Market share and costs of biologic therapies for inflammatory bowel disease in the USA. Aliment Pharmacol Ther. 2018;47:364–70. 29164650 10.1111/apt.14430 PMC5760274

[12] Kimura H , Takahashi K , Futami K , Ikeuchi H , Tatsumi K , Watanabe K , et al. Has widespread use of biologic and immunosuppressant therapy for ulcerative colitis affected surgical trends? Results of a questionnaire survey of surgical institutions in Japan. Surg Today. 2016;46:930–8. 26467558 10.1007/s00595-015-1259-3

[13] Uchino M , Ikeuchi H , Hata K , Okada S , Ishihara S , Morimoto K , et al. Changes in the rate of and trends in colectomy for ulcerative colitis during the era of biologics and calcineurin inhibitors based on a Japanese nationwide cohort study. Surg Today. 2019;49(12):1066‐73. 31309329 10.1007/s00595-019-01845-2

[14] Varma S , Hu J , Mehta A . Initial medical and surgical management of inflammatory bowel disease in the biologic era: a comparison between the United States and China. JGH Open. 2019;3(3):234–41. 31276042 10.1002/jgh3.12146 PMC6586599

[15] Rutgeerts PJ , Sandborn WJ , Feagan BG , Reinisch W , Olson A , Johanns J , et al. Infliximab for induction and maintenance therapy for ulcerative colitis. N Engl J Med. 2005;355:2462–76. 10.1056/NEJMoa050516 16339095

[16] Sandborn WJ , Rutgeerts P , Feagan BG , Reinisch W , Olson A , Johanns J , et al. Colectomy rate comparison after treatment of ulcerative colitis with placebo or infliximab. Gastroenterology. 2009;137:1250–60. 19596014 10.1053/j.gastro.2009.06.061

[17] Jarnerot G , Hertervig E , Friis‐Liby I , Blomquist L , Karlén P , Grännö C , et al. Infliximab as rescue therapy in severe to moderately severe ulcerative colitis: a randomized, placebo‐controlled study. Gastroenterlogy. 2005;128:1805–11. 10.1053/j.gastro.2005.03.003 15940615

[18] Gustavsson A , Järnerot G , Hertervig E , Friis‐Liby I , Blomquist L , Karlén P , et al. Clinical trial: colectomy after rescue therapy in ulcerative colitis—3‐year follow‐up of the Swedish‐Danish controlled infliximab study. Aliment Pharmacol Ther. 2010;32:984–9. 20937043 10.1111/j.1365-2036.2010.04435.x

[19] Williams JG , Alam FM , Alrubaiy L , Clement C , Cohen D , Grey M , et al. Comparison of infliximab and ciclosporin in steroid resistant ulcerative colitis: pragmatic randomised trial and economic evaluation (CONSTRUCT). Health Technol Assess. 2016;20:1–320. 10.3310/hta20440 PMC4939417 27329657

[20] Laharie D , Bourreille A , Branche J , Allez M , Bouhnik Y , Filippi J , et al. Ciclosporin versus infliximab in patients with severe ulcerative colitis refractory to intravenous steroids: a parallel, open‐label randomised controlled trial. Lancet. 2012;380:1909–15. 23063316 10.1016/S0140-6736(12)61084-8

[21] Laharie D , Bourreille A , Branche J , Allez M , Bouhnik Y , Filippi J , et al. Long‐term outcome of patients with steroid‐refractory acute severe UC treated with ciclosporin or infliximab. Gut. 2018;67:237–43. 28053054 10.1136/gutjnl-2016-313060

[22] Liu YJ , Fan H , Zhen WW , Yu X , Chen JT , Wang CD . Pooled analysis of the comparative efficacy between tacrolimus and infliximab for ulcerative colitis. Medicine (Baltimore). 2018;97(32):e11440. 30095612 10.1097/MD.0000000000011440 PMC6133612

[23] Narula N , Fine M , Colombel JF , Marshall JK , Reinisch W . Systematic review: sequential rescue therapy in severe ulcerative colitis: do the benefits outweigh the risks? Inflamm Bowel Dis. 2015;21:1683–94. 25839775 10.1097/MIB.0000000000000350

[24] Takeuchi K , Shimoyama T , Yamamoto T . Comparison of safety and efficacy of tacrolimus versus infliximab for active ulcerative colitis. Dig Dis. 2018;36:106–12. 29050007 10.1159/000481815

[25] Abelson JS , Michelassi F , Mao J , Sedrakyan A , Yeo H . Higher surgical morbidity for ulcerative colitis patients in the era of biologics. Ann Surg. 2018;268:311–7. 28448381 10.1097/SLA.0000000000002275

[26] Shinagawa T , Hata K , Ikeuchi H , Fukushima K , Futami K , Sugita A , et al. Rate of reoperation decreased significantly after year 2002 in patients with Crohn's disease. Clin Gastroenterol Hepatol. 2019 10.1016/j.cgh.2019.07.025 31336198

[27] Bedrikovetski S , Dudi‐Venkata N , Kroon HM , Liu J , Andrews JM , Lewis M , et al. Systematic review of rectal stump management during and after emergency total colectomy for acute severe ulcerative colitis. ANZ J Surg. 2019 10.1111/ans.15075 30919553

[28] Macaluso FS , Cavallaro F , Felice C , Mazza M , Armuzzi A , Gionchetti P , et al. Risk factors and timing for colectomy in chronically active refractory ulcerative colitis: a systematic review. Dig Liver Dis. 2019;51:613–20. 30826279 10.1016/j.dld.2019.01.018

[29] Laureti S , Gionchetti P , Cappelli A , Vittori L , Contedini F , Rizzello F , et al. Refractory complex Crohn's perianal fistulas: a role for autologous microfragmented adipose tissue injection. Inflamm Bowel Dis. 2019 10.1093/ibd/izz051 PMC6943693 31220252

[30] Gutiérrez A , Rivero M , Martín‐Arranz MD , García Sánchez V , Castro M , Barrio J , et al. Perioperative management and early complications after intestinal resection with ileocolonic anastomosis in Crohn's disease: analysis from the PRACTICROHN study. Gastroenterol Rep (Oxf). 2019;7:168–75. 31217980 10.1093/gastro/goz010 PMC6573802

[31] Allez M , Auzolle C , Ngollo M , Bottois H , Chardiny V , Corraliza AM , et al. T cell clonal expansions in ileal Crohn's disease are associated with smoking behaviour and postoperative recurrence. Gut. 2019;68(11):1961‐70. 30792246 10.1136/gutjnl-2018-317878

[32] Law CCY , Narula A , Lightner AL , McKenna NP , Colombel J‐F , Narula N . Systematic review and meta‐analysis: preoperative vedolizumab treatment and postoperative complications in patients with inflammatory bowel disease. J Crohns Colitis. 2018;12:538–45. 29718245 10.1093/ecco-jcc/jjy022

[33] Yung DE , Horesh N , Lightner AL , Ben‐Horin S , Eliakim R , Koulaouzidis A , et al. Systematic review and meta‐analysis: vedolizumab and postoperative complications in inflammatory bowel disease. Inflamm Bowel Dis. 2018;24:2327–38. 29788385 10.1093/ibd/izy156

[34] Novello M , Stocchi L , Holubar S , Shawki S , Lipman J , Gorgun E , et al. Surgical outcomes of patients treated with ustekinumab vs. vedolizumab in inflammatory bowel disease: a matched case analysis. Int J Colorectal Dis. 2019;34:451–7. 30535559 10.1007/s00384-018-3212-6

[35] Novello M , Stocchi L , Steele SR , Holubar SD , Duraes LC , Kessler H , et al. Case‐matched comparison of postoperative outcomes following surgery for inflammatory bowel disease after exposure to vedolizumab vs. other biologics. J Crohns Colitis. 2019 10.1093/ecco-jcc/jjz129 31328222

[36] Kopylov U , Ben‐Horin S , Zmora O , Eliakim R , Katz LH . Anti‐tumor necrosis factor and postoperative complications in Crohn's disease: systematic review and meta‐analysis. Inflamm Bowel Dis. 2012;18:2404–13. 22467533 10.1002/ibd.22954

[37] Billioud V , Ford AC , Tedesco ED , Colombel JF , Roblin X , Peyrin‐Biroulet L . Preoperative use of anti‐TNF therapy and postoperative complications in inflammatory bowel diseases: a meta‐analysis. J Crohns Colitis. 2013;7:853–67. 23523418 10.1016/j.crohns.2013.01.014

[38] Lau C , Dubinsky M , Melmed G , Vasiliauskas E , Berel D , McGovern D . The impact of preoperative serum anti‐TNFa therapy levels on early postoperative outcomes in inflammatory bowel disease surgery. Ann Surg. 2015;261:487–96. 24950263 10.1097/SLA.0000000000000757 PMC4994811

[39] Yamada A , Komaki Y , Patel N , Komaki F , Aelvoet AS , Tran AL , et al. Risk of postoperative complications among inflammatory bowel disease patients treated preoperatively with vedolizumab. Am J Gastroenterol. 2017;112:1423–9. 28719595 10.1038/ajg.2017.201

[40] Fumery M , Seksik P , Auzolle C , Munoz‐Bongrand N , Gornet J‐M , Boschetti G , et al. Postoperative complications after ileocecal resection in Crohn's disease: a prospective study from the REMIND group. Am J Gastroenterol. 2017;112:337–45. 27958285 10.1038/ajg.2016.541

[41] Kulaylat A , Kulaylat AN , Schaefer EW , Tinsley A , Williams E , Koltun W , et al. Association of preoperative anti‐tumor necrosis factor therapy with adverse postoperative outcomes in patients undergoing abdominal surgery for ulcerative colitis. JAMA Surg. 2017;152:e171538. 28614561 10.1001/jamasurg.2017.1538 PMC5831468

[42] Lightner AL , McKenna NP , Moncrief S , Pemberton JH , Raffals LE , Mathis KL . Surgical outcomes in vedolizumab‐treated patients with ulcerative colitis. Inflamm Bowel Dis. 2017;23:2197–201. 28858072 10.1097/MIB.0000000000001248

[43] Sarlos P , Szemes K , Hegyi P , Garami A , Szabo I , Illes A , et al. Steroid but not biological therapy elevates the risk of venous thromboembolic events in inflammatory bowel disease: a meta‐analysis. J Crohns Colitis. 2018;12:489–98. 29220427 10.1093/ecco-jcc/jjx162

[44] Benlice C , Holubar SD , Gorgun E , Stocchi L , Lipman JM , Kalady MF , et al. Extended venous thromboembolism prophylaxis after elective surgery for IBD patients: nomogram‐based risk assessment and prediction from nationwide cohort. Dis Colon Rectum. 2018;61:1170–9. 30192325 10.1097/DCR.0000000000001189

[45] Nguyen GC , Du L , Chong RY , Jackson TD . Hypoalbuminaemia and postoperative outcomes in inflammatory bowel disease: the NSQIP Surgical Cohort. J Crohns Colitis. 2019;13(11):1433‐8. 31253985 10.1093/ecco-jcc/jjz083 PMC6821313

[46] de Groof EJ , Stevens TW , Eshuis EJ , Gardenbroek TJ , Bosmans JE , van Dongen JM , et al. Cost‐effectiveness of laparoscopic ileocaecal resection versus infliximab treatment of terminal ileitis in Crohn's disease: the LIR!C Trial. Gut. 2019;68(10):1774‐1780. 31233395 10.1136/gutjnl-2018-317539

[47] Murthy SK , Begum J , Benchimol EI , Bernstein CN , Kaplan GG , McCurdy JD , et al. Introduction of anti‐TNF therapy has not yielded expected declines in hospitalisation and intestinal resection rates in inflammatory bowel diseases: a population‐based interrupted time series study. Gut. 2019 10.1136/gutjnl-2019-318440 PMC6984056 31196874

[48] Liska D , Bora Cengiz T , Novello M , Aiello A , Stocchi L , Hull TL , et al. Do patients with inflammatory bowel disease benefit from an enhanced recovery pathway? Inflamm Bowel Dis. 2019 10.1093/ibd/izz172 31372647

[49] Schwartzberg DM , Remzi FH . The role of laparoscopic, robotic, and open surgery in uncomplicated and complicated inflammatory bowel disease. Gastrointest Endosc Clin N Am. 2019;29:563–76. 31078253 10.1016/j.giec.2019.02.012

[50] Mizushima T , Nakajima K , Takeyama H , Naito A , Osawa H , Uemura M , et al. Single‐incision laparoscopic surgery for stricturing and penetrating Crohn's disease. Surg Today. 2016;46:203–8. 25735739 10.1007/s00595-015-1145-z

[51] Lightner AL , Grass F , McKenna NP , Tilman M , Alsughayer A , Kelley SR , et al. Short‐term postoperative outcomes following robotic versus laparoscopic ileal pouch‐anal anastomosis are equivalent. Tech Coloproctol. 2019;23:259–66. 30941619 10.1007/s10151-019-01953-8

[52] Cañete F , Mañosa M , Clos A , Cabré E , Domènech E . Review article: the relationship between obesity, bariatric surgery, and inflammatory bowel disease. Aliment Pharmacol Ther. 2018;48:807–16. 30178869 10.1111/apt.14956

[53] Heshmati K , Lo T , Tavakkoli A , Sheu E . Short‐term outcomes of inflammatory bowel disease after Roux‐en‐Y gastric bypass vs sleeve gastrectomy. J Am Coll Surg. 2019;228:893–901. 30797083 10.1016/j.jamcollsurg.2019.01.021

[54] An SB , Kim BC , Kim JY , Kim JW , Lee SJ . Results of Laparotomy and Laparoscopy for Perforated Colonic Diverticulitis. JSLS. 2019;23(3):e2019. 10.4293/JSLS.2019.00007 PMC6687474 31431798

[55] Patel R , Zagadailov P , Merchant AM . Laparoscopic colectomy for diverticulitis in patients with pre‐operative respiratory comorbidity: analysis of post‐operative outcomes in the United States from 2005 to 2017. Surg Endosc. 2019 10.1007/s00464-019-06943-3 31286256

[56] Ahmed AM , Moahammed AT , Mattar OM , Mohamed EM , Faraag EA , AlSafadi AM , et al. Surgical treatment of diverticulitis and its complications: a systematic review and meta‐analysis of randomized control trials. Surgeon. 2018;16:372–83. 30033140 10.1016/j.surge.2018.03.011

[57] Galetin A , Rink AD , Vestweber B , Vestweber KH , Galetin T . Single‐incision laparoscopic versus open sigmoidectomy for diverticular disease: a disease‐stratified matched‐pair analysis. Dig Surg. 2019 10.1159/000497449 30921802

[58] Ogilvie JW Jr , Saunders RN , Parker J , Luchtefeld MA . Sigmoidectomy for diverticulitis. A propensity‐matched comparison of minimally invasive approaches. J Surg Res. 2019;243:434–9. 31279270 10.1016/j.jss.2019.06.018

[59] Al‐Temimi MH , Chandrasekaran B , Agapian J , Peters WR Jr , Wells KO . Robotic versus laparoscopic elective colectomy for left side diverticulitis: a propensity score‐matched analysis of the NSQIP database. Int J Colorectal Dis. 2019;34:1385–92. 31230107 10.1007/s00384-019-03334-x

[60] Cassini D , Depalma N , Grieco M , Cirocchi R , Manoochehri F , Baldazzi G . Robotic pelvic dissection as surgical treatment of complicated diverticulitis in elective settings: a comparative study with fully laparoscopic procedure. Surg Endosc. 2019;33:2583–90. 30406387 10.1007/s00464-018-6553-x

[61] Raskin ER , Keller DS , Gorrepati ML , Akiel‐Fu S , Mehendale S , Cleary RK . Propensity‐matched analysis of sigmoidectomies for diverticular disease. JSLS. 2019;23(1):e2018. 10.4293/JSLS.2018.00073 PMC6328361 30675092

[62] Yeom JH , Lee JH , Song JS , Lee MH , Kim MG . Extending the Indication for laparoscopic surgery in patients with pan‐peritonitis. Surg Laparosc Endosc Percutan Tech. 2019;29:120–5. 30531448 10.1097/SLE.0000000000000613

[63] Maggard MA , Zingmond D , O'Connell JB , Ko CY . What proportion of patients with an ostomy (for diverticulitis) get reversed? Am Surg. 2004;70:928–31. 15529854

[64] Vermeulen J , Coene PPLO , Van Hout NM , van der Harst E , Gosselink MP , Mannaerts GHH , et al. Restoration of bowel continuity after surgery for acute perforated diverticulitis: should Hartmann's procedure be considered a one‐stage procedure? Colorectal Dis. 2006;11:619–24. 10.1111/j.1463-1318.2008.01667.x 18727727

[65] Lambrichts DPV , Vennix S , Musters GD , Mulder IM , Swank HA , Hoofwijk AGM , et al. Hartmann's procedure versus sigmoidectomy with primary anastomosis for perforated diverticulitis with purulent or faecal peritonitis (LADIES): a multicentre, parallel‐group, randomised, open‐label, superiority trial. Lancet Gastroenterol Hepatol. 2019;4:599–610. 31178342 10.1016/S2468-1253(19)30174-8

[66] Lee JM , Bai P , Chang J , El Hechi M , Kongkaewpaisan N , Bonde A , et al. Hartmann's procedure vs primary anastomosis with diverting loop ileostomy for acute diverticulitis: nationwide analysis of 2,729 emergency surgery patients. J Am Coll Surg. 2019;229:48–55. 30902639 10.1016/j.jamcollsurg.2019.03.007

[67] Ahmadi N , Howden WB , Ahmadi N , Byrne CM , Young CJ . Increasing primary anastomosis rate over time for the operative management of acute diverticulitis. ANZ J Surg. 2019;89(9):1080–84 31272133 10.1111/ans.15321

[68] Cauley CE , Patel R , Bordeianou L . Use of primary anastomosis with diverting ileostomy in patients with acute diverticulitis requiring urgent operative intervention. Dis Colon Rectum. 2018;61:586–92. 29630003 10.1097/DCR.0000000000001080

[69] Cirocchi R , Afshar S , Shaban F , Nascimbeni R , Vettoretto N , Di Saverio S , et al. Perforated sigmoid diverticulitis: Hartmann's procedure or resection with primary anastomosis‐a systematic review and meta‐analysis of randomised control trials. Tech Coloproctol. 2018;22:743–53. 29995173 10.1007/s10151-018-1819-9

[70] Goldstone RN , Cauley CE , Chang DC , Kunitake H , Ricciardi R , Bordeianou L . The effect of surgical training and operative approach on outcomes in acute diverticulitis: should guidelines be revised? Dis Colon Rectum. 2019;62(1):71–8. 30451762 10.1097/DCR.0000000000001240

[71] Acuna SA , Wood T , Chesney TR , Dossa F , Wexner SD , Quereshy FA , et al. Operative strategies for perforated diverticulitis: a systematic review and meta‐analysis. Dis Colon Rectum. 2018;61:1442–53. 30371549 10.1097/DCR.0000000000001149

[72] Kohl A , Rosenberg J , Bock D , Bisgaard T , Skullman S , Thornell A , et al. Two‐year results of the randomized clinical trial DILALA comparing laparoscopic lavage with resection as treatment for perforated diverticulitis. Br J Surg. 2018;105:1128–34. 29663316 10.1002/bjs.10839 PMC6055876

[73] Penna M , Markar SR , Mackenzie H , Hompes R , Cunningham C . Laparoscopic lavage versus primary resection for acute perforated diverticulitis: review and meta‐analysis. Ann Surg. 2018;267(2):252–8. 28338510 10.1097/SLA.0000000000002236

[74] Beyer‐Berjot L , Maggiori L , Loiseau D , De Korwin J‐D , Bongiovanni J‐P , Lesprit P , et al. Emergency surgery in acute diverticulitis: a systematic review. Dis Colon Rectum. 2019 10.1097/DCR.0000000000001327 30694823

[75] Sneiders D , Lambrichts DPV , Swank HA , Blanken‐Peeters CFJM , Nienhuijs SW , Govaert MJPM , et al. Long‐term follow‐up of a multicentre cohort study on laparoscopic peritoneal lavage for perforated diverticulitis. Colorectal Dis. 2019;21:705–14. 30771246 10.1111/codi.14586 PMC6850083

[76] Resio BJ , Jean R , Chiu AS , Pei KY . Association of timing of colostomy reversal with outcomes following Hartmann procedure for diverticulitis. JAMA Surg. 2019;154:218–24. 30476948 10.1001/jamasurg.2018.4359 PMC6439630

[77] Benlice C , Delaney CP , Liska D , Hrabe J , Steele S , Gorgun E . Individual surgeon practice is the most important factor influencing diverting loop ileostomy creation for patients undergoing sigmoid colectomy for diverticulitis. Am J Surg. 2018;215:442–5. 29153979 10.1016/j.amjsurg.2017.10.046

[78] Luu LH , Vuong NL , Yen VTH , Phuong DTT , Vu BK , Thanh NV , et al. Laparoscopic diverticulectomy versus non‐operative treatment for uncomplicated right colonic diverticulitis. Surg Endosc. 2019 10.1007/s00464-019-06981-x 31309310

[79] Bolkenstein HE , Consten ECJ , van der Palen J , van de Wall BJM , Broeders IAMJ , Bemelman WA , et al. Long‐term outcome of surgery versus conservative management for recurrent and ongoing complaints after an episode of diverticulitis: 5‐year follow‐up results of a multicenter randomized controlled trial (DIRECT‐trial). Ann Surg. 2019;269:612–20. 30247329 10.1097/SLA.0000000000003033

[80] Bolkenstein HE , de Wit GA , Consten ECJ , Van de Wall BJM , Broeders IAMJ , Draaisma WA . Cost‐effectiveness analysis of a multicentre randomized clinical trial comparing surgery with conservative management for recurrent and ongoing diverticulitis (DIRECT trial). Br J Surg. 2019;106:448–57. 30566245 10.1002/bjs.11024

[81] Valizadeh N , Suradkar K , Kiran RP . Specific factors predict the risk for urgent and emergent colectomy in patients undergoing surgery for diverticulitis. Am Surg. 2018;84:1781–6. 30747633

[82] Varma S , Mehta A , Canner JK , Azar F , Efron DT , Efron J , et al. Surgery after an initial episode of uncomplicated diverticulitis: does time to resection matter? J Surg Res. 2019;234:224–30. 30527478 10.1016/j.jss.2018.09.043

[83] Lambrichts DPV , Bolkenstein HE , van der Does DCHE , Dieleman D , Crolla RMPH , Dekker JWT , et al. Multicentre study of non‐surgical management of diverticulitis with abscess formation. Br J Surg. 2019;106:458–66. 30811050 10.1002/bjs.11129 PMC6593757

[84] Jehan F , Zeeshan M , Con J , Hanna K , Tang A , Hamidi M , et al. Metabolic syndrome exponentially increases the risk of adverse outcomes in operative diverticulitis. J Surg Res. 2019;245:544–51. 31470335 10.1016/j.jss.2019.07.075

[85] Bordeianou L , Cauley CE , Patel R , Bleday R , Mahmood S , Kennedy K , et al. Prospective creation and validation of the PREVENTT (Prediction and Enaction of Prevention Treatments Trigger) Scale for Surgical Site Infections (SSIs) in patients with diverticulitis. Ann Surg. 2019;270(6):1124–30. 29916880 10.1097/SLA.0000000000002859

[86] Al‐Temimi MH , Trujillo CN , Mahlberg S , Ruan J , Nguyen P , Yuhan R , et al. Surgical intervention for right‐side diverticulitis: a case‐matched comparison with left‐side diverticulitis. Am Surg. 2018;84:1608–12. 30747679

